# The long-term in vivo behavior of polymethyl methacrylate bone cement in total hip arthroplasty

**DOI:** 10.3109/17453674.2011.625538

**Published:** 2011-11-24

**Authors:** Hiroyuki Oonishi, Haruhiko Akiyama, Mitsuru Takemoto, Toshiyuki Kawai, Koji Yamamoto, Takao Yamamuro, Hironobu Oonishi, Takashi Nakamura

**Affiliations:** ^1^H. Oonishi Memorial Joint Replacement Institute, Tominaga Hospital, Osaka; ^2^Department of Orthopaedics, Kyoto University, Kyoto; ^3^Research Institute for Production Development, Kyoto, Japan

## Abstract

**Background and purpose:**

The long-term success of cemented total hip arthroplasty (THA) has been well established. Improved outcomes, both radiographically and clinically, have resulted mainly from advances in stem design and improvements in operating techniques. However, there is concern about the durability of bone cement in vivo. We evaluated the physical and chemical properties of CMW1 bone cements retrieved from patients undergoing revision THA.

**Methods:**

CMW1 cements were retrieved from 14 patients who underwent acetabular revision because of aseptic loosening. The time in vivo before revision was 7–30 years. The bending properties of the retrieved bone cement were assessed using the three-point bending method. The molecular weight and chemical structure were analyzed by gel permeation chromatography and Fourier-transform infrared spectroscopy. The porosity of the bone cements was evaluated by 3-D microcomputer tomography.

**Results:**

The bending strength decreased with increasing time in vivo and depended on the density of the bone cement, which we assume to be determined by the porosity. There was no correlation between molecular weight and time in vivo. The infrared spectra were similar in the retrieved cements and in the control CMW1 cements.

**Interpretation:**

Our results indicate that polymer chain scission and significant hydrolysis do not occur in CMW1 cement after implantation in vivo, even in the long term. CMW1 cement was stable through long-term implantation and functional loading.

The concept behind Charnley low-friction arthroplasty was established in the 1960s, and the fundamental principles have remained unchanged since then. Several clinical studies have recently reported the long-term success of total hip arthroplasty (THA). Wroblewski et al. (2009) reported good results using Charnley low-friction arthroplasty with a follow-up of 30–40 years. Overall, 90% of hips were free from pain, and activity was normal in 59% of the patients. Carrington et al. (2009) reported the results of the Exeter Universal cemented femoral component after 15–17 years. With an endpoint of revision for aseptic loosening, the survivorship at 17 years was 100% for the femoral component and 90% for the acetabular component. With all reasons for reoperation as the endpoint, the survivorship was 81%. A variety of cemented stems designed according to various concepts have been used, and several improvements have been incorporated into the operating techniques (Madey et al. 1997, Noble et al. 1998, Scheerlinck and Casteleyn 2006). Although self-curing polymethyl methacrylate (PMMA) bone cements have been used for fixation of the implants for the past 50 years, the composition of the cements has remained essentially unaltered. The ultra-long clinical and radiographic success of cemented THA may depend on the mechanical and chemical longevity of the bone cements in vivo.

Several authors have reported on the in vivo behavior of PMMA bone cement in the implanted joint. Some studies have shown aging of PMMA in vivo. Hughes et al. (2003) showed a decrease in molecular weight and hydrolysis of PMMA associated with long-term implantation. Looney and Park (1986) reported a reduction in flexural strength but not in compressive strength. Fernandez-Fairen and Vazquez (1983) analyzed the compressive properties of the retrieved CMW1 cements and found a decrease in the compressive modulus and strength after long implantation periods. By contrast, Ries et al. (2006) concluded that the most important factor for the mechanical properties of bone cement in vivo is not the implant duration but the porosity. It remains unknown whether the mechanical and chemical properties of bone cement change in vivo, and how these changes affect the long-term outcome of cemented THA. We investigated various properties, including molecular weight, chemical structure, bending properties, density, and porosity in retrieved bone cements.

## Patients and methods

### Sample preparation

CMW1 cements were retrieved from 14 patients who underwent acetabular revision because of aseptic loosening. The median time in vivo before revision was 15 (7–30) years. The retrieved samples were rinsed in saline solution and ethanol, and then stored at room temperature until they were examined.

### Molecular weight analysis

The average molecular weight and the molecular weight distribution of the retrieved cements were assessed by gel permeation chromatography (GPC). Molecular weight calibration was established based on polystyrene standards. Briefly, samples were dissolved in tetrahydrofuran (THF) to a concentration of 2 mg/mL at room temperature, and then filtered through 0.45-μm disk filters. Each sample was injected into a 30-cm long GPC gel column (Shodex, Tokyo, Japan) with an inner diameter of 8.0 mm, which was packed with THF with a pore size of 1,000 nm. The injection volume was 50 μL and the flow rate was 1 mL/min at 40°C. A differential refractive index detector (Hitachi L-2000; Hitachi, Tokyo, Japan) was used to monitor changes in the concentration of the sample. The molecular weight distributions of the samples relative to polystyrene were found in terms of the number-averaged molecular weight M_n_, the weight-averaged molecular weight M_w_, and the polydispersity index (PDI; M_w_/M_n_ ratio).

### Fourier-transform infrared spectroscopy (FTIR) analysis

Chemical analysis was performed using FTIR. The FTIR spectra were obtained using a Spectrum BX spectrometer (PerkinElmer, Waltham, MA). All transmission spectra were collected with a spectral resolution of 4 cm^–1^ and spectral range of 4000 to 600 cm^–1^ using KBr pellets. Unimplanted control CMW1 cement specimens were freshly prepared by mixing the powder and liquid components by hand according to the manufacturer's instructions. They were then sent for FTIR analysis within 1 month of preparation.

### Bending properties

The retrieved cement specimens were cut and scraped into rectangular specimens (20 mm × 4 mm × 3 mm) using a rotational scraping machine (BUEHLER EcoMet 3000; BUEHLER Ltd., Lake Bluff, IL) with 1–9 specimens for each sample. The bending strength and bending modulus of each retrieved cement specimen and each freshly prepared CMW1 cement specimen were analyzed using an Instron 5500 instrument (Instron, Norwood, MA) at 23 ± 1°C. The crosshead speed and the span were 0.5 mm/min and 15 mm, respectively, when using the 3-point bending method. The values for the bending modulus were derived from the stress-strain curves obtained from the bending tests, as described previously (Shinzato et al. 2002).

### Porosity analysis

A microfocus X-ray computed tomography system (SMX-100CT-SV3; Shimadzu Co., Kyoto, Japan) was used to acquire microstructural information from the retrieved cements. The entire set of radiographs was deconvoluted by computer software to reconstruct a 3-D image of the microstructure with a voxel size of 16 μm^3^. The 3-D data were processed with commercially available 3-D image-processing software (VG Studio MAX 2.0; Volume Graphics, Heidelberg, Germany), and the porosity of the retrieved cements was calculated from the binary material images. The spatial boundary between the pores and the cement was established easily because of the large differences in density.

### Statistics

Data from each test were compared by analysis of variance (ANOVA) to determine the overall significance of data trends. For all analyses, p < 0.01 was considered significant.

## Results

**Figure 1:**
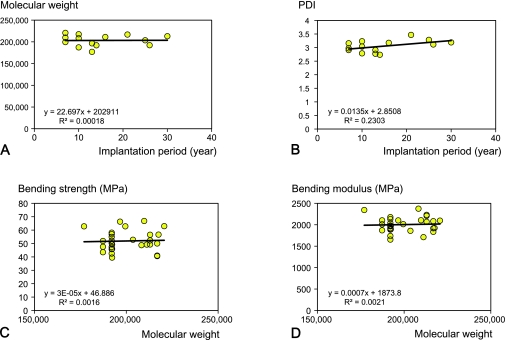
A. Relationship between implantation period and molecular weight. B. Relationship between implantation period and PDI. C. Relationship between molecular weight and bending strength. D. Relationship between molecular weight and bending modulus.

**Figure 2.**
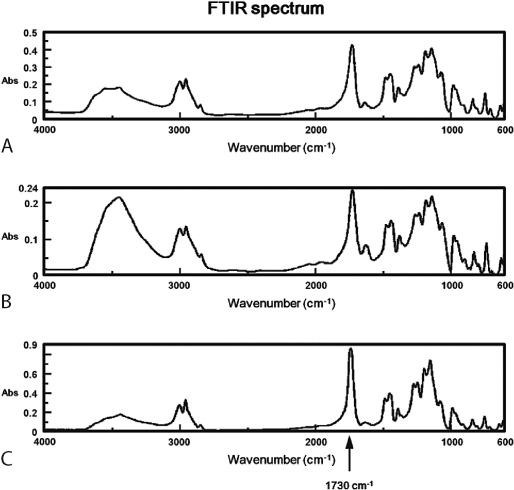
FTIR spectrum of control CMW1 cement (A) and CMW1 cement retrieved 16 years (B) and 30 years (C) after implantation.

The M_w_ of the samples ranged from 170,000 to 220,000 g/mol (Table). The molecular weight and PDI did not correlate with the time in vivo (r = 0.013, p = 1.0; r = 0.54, p = 0.05, respectively) (Figure 1A and B). Because the molecular weight of the polymer is proportional to the degree of polymerization of the monomer unit, this result suggested that scission of polymer chains did not occur in CMW1 cement in vivo. There was no substantial difference in FTIR spectra between the CMW1 cements in fresh samples after they were cured and in samples retrieved 16 and 30 years after implantation (Figure 2). The spectra of methyl methacrylate homopolymer showed a distinctive absorbance band around 1,730 cm^–1^, corresponding to the C=O stretch of the ester group (Hughes et al. 2003). This distinctive absorbance band did not differ between freshly prepared and retrieved CMW1 cements. These results indicate that no substantial hydrolysis of the ester group occurred in CMW1 cement in vivo, even after many years.

**Table:** Summary of molecular weights for the retrieved cements

Sample no.	Implantation time (years)	M_w_(g/mol)	M_n_(g/mol)	PDI
1	7	209,737	66,412	3.16
2	7	199,478	68,608	2.91
3	7	220,660	74,217	2.97
4	10	187,296	67,205	2.79
5	10	208,271	64,416	3.23
6	10	217,552	71,617	3.04
7	13	176,996	63,839	2.77
8	13	196,600	67,618	2.91
9	14	192,056	70,209	2.74
10	16	211,157	66,509	3.17
11	21	216,827	62,554	3.47
12	25	203,630	62,005	3.28
13	26	192,270	61,753	3.11
14	30	212,962	66,750	3.19

**Figure 3.**
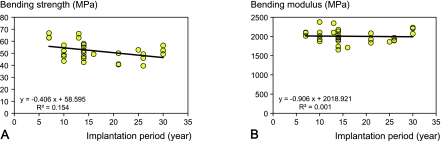
A. Relationship between implantation period and bending strength. B. Relationship between implantation period and bending modulus.

The molecular weight was not related to the bending strength or the bending modulus (r = 0.040, p = 0.83; r = 0.046, p = 0.8, respectively) (Figure 1C and D). By contrast, the bending strength of each cement specimen was reduced with increasing time in vivo, but this was not statistically significant (r = –0.39, p = 0.03) (Figure 3A). In addition, there was no correlation between bending modulus and the length of the implantation period (r = –0.038, p = 0.8) (Figure 3B).

**Figure 4.**
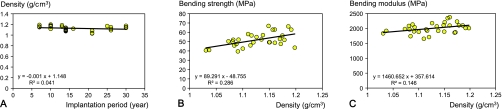
A. Relationship between implantation period and density. B. Relationship between density and bending strength. C. Relationship between density and bending modulus.

**Figure 5.**
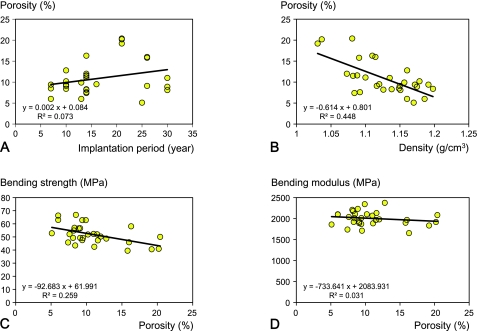
A. Relationship between implantation period and porosity. B. Relationship between density and porosity. C. Relationship between porosity and bending strength. D. Relationship between porosity and bending modulus.

The density of the specimens was calculated from their dimension and weight. There was no correlation between the density of the cements and length of time in vivo (r = –0.20, p = 0.3) (Figure 4A). Density was found to be strongly correlated to bending strength (r = 0.54, p = 0.002) but not to bending modulus (r = 0.38, p = 0.04) (Figure 4B and C). There was no correlation between the porosity of the specimens and the time in vivo (r = 0.27, p = 0.1) (Figure 5A). There was a strong correlation between the density and the porosity of the cement (r = –0.67, p < 0.001) (Figure 5B). The porosity correlated with bending strength (r = –0.51, p = 0.004) (Figure 5C) but not with bending modulus (r = –0.18, p = 0.4) (Figure 5D). These results indicate that the porosity of the bone cement defined the in vivo mechanical properties of the retrieved specimens. As the porosity of cement is unlikely to change substantially after curing, the mechanical properties of the cement should remain stable.

## Discussion

One of the factors that dictate the long-term stability of cemented stems is the longevity of the cement itself. Failure of the cemented stem implanted in THAs is induced by excessive stress in the cement mantle, leading to micromovement of the stem and debonding at the cement-stem interface, along with microcracking in the cement (Gardiner and Hozack 1994, Ong et al. 2002). This cement damage finally causes stem loosening, stem subsidence, increased production of wear particles, and osteolysis. Thus, retention of the chemical and mechanical properties of the bone cement in vivo is critical for achievment of long-term success in THA.

Although previous studies have used various tests to investigate the in vivo behavior and chemical and mechanical properties of bone cements, there is no consensus on the aging of the cement (Fernandez-Fairen and Vazquez 1983, Looney and Park 1986, Hughes et al. 2003, Ries et al. 2006). The molecular structure, the dispersity of the contrast medium, or the distribution of pores introduced during the preparation of bone cements may affect their mechanical properties, and these complex factors make interpretation of the data—and comparison between studies—difficult.

The mechanical properties of implanted bone cements depend on the chemistry of the bone cement and on the mixing method (Lewis 1997). Our study shows that there is no correlation between the molecular weight of the bone cement and the time in vivo, and that neither degradation of the main chain nor hydrolysis of PMMA occurs in CMW1 cement. We also found no relationship between molecular weight and bending strength or bending modulus. Our results contrast with those of Hughes et al. (2003), who found a decrease in molecular weight and chemical degradation in retrieved Simplex P and Palacos R cements that had aged up to 23 years in vivo. CMW1 powder comprises only methyl methacrylate, whereas Palacos R powder contains methylacrylate, which is polar and hydrophilic. Simplex P contains hydrophobic styrene comonomer, which has no polarity. In vivo degradation of bone cements is related to particular combinations of localized acidic pH, free radical oxidation induced by superoxidizing substances, and hydrolyzing enzymes. One possible explanation of these results (Hughes et al. 2003) that the differences in chemical properties of bone cements confer different sensitivities to biological processes that induce degradation. The cements retrieved from total knee arthroplasties after similar in vivo aging times to those from THAs showed little change in their structural properties, suggesting that in vivo degradation of the bone cements is related to the biological response to the implant and the local environment of the joint (Hughes et al. 2003). These lines of evidence strongly suggest that the degradation of bone cements in vivo depends partly on the environment of implantation and partly on the composition of bone cements. In addition, the FTIR spectra showed an increase in the large band at 3,500 cm-1, attributable to OH stretching in water, in some retrieved specimens. Water is a plasticizer for methacrylate, and could therefore reduce the mechanical properties of bone cement. This should be clarified by further studies.

Although Dall et al. (2007) reported that inter-batch and intra-batch variability was seen in the viscosity of all brands of bone cement, the strong relationship between bending strength and density or porosity shows that the mechanical properties of bone cements depend on their density or porosity (Weinstein et al. 1976, Wang et al. 1993, Chaplin et al. 2006). Our study and studies of others have shown significant correlations between density and porosity. High porosity contributes to microcracks in the bone cement, which lead to release of PMMA particles and induce aseptic loosening and osteolysis (James et al. 1992, Graham et al. 2003, Hoey et al. 2009). In addition, cracks and voids could be also generated by micromovement between cement and prosthesis/bone or by wear, and may affect the mechanical properties. Thus, the porosity of bone cement is a critical factor in determining the mechanical properties of the bone cement in vivo. The porosity of bone cement is determined by the method used to prepare and apply it. In the modern cementing technique, vacuum mixing reduces the porosity of bone cement and improves its fatigue resistance (James et al. 1992, Wang et al. 1993). Graham et al. (2000) reported that the internal porosity of bone cement is greatly reduced by vacuum mixing, whereas a higher porosity introduced during the hand-mixing process caused all hand-mixed specimens to have inferior fracture and fatigue resistance to their vacuum-mixing counterparts. Moreover, elevated pressure during curing helps reduce the porosity of the bone cement; thus, high-pressure insertion of implants substantially improves the mechanical properties of the bone cement (Bayne et al. 1975, Apostolou et al. 2007). Taken together, this evidence shows that the mechanical properties of implanted bone cement depend on the operating techniques rather than the period of implantation.

Ries et al. (2006) investigated the variables fracture toughness, porosity, molecular weight, and time in vivo of the bone cement, and concluded that porosity and fracture toughness are significantly and inversely related. All bone cements retrieved in the acetabular reconstruction in our study were hand-mixed and were applied to the acetabulum without use of a cement pressurizer. One possible reason that the porosity increases in relation to the time in vivo is that cement pressurization in the implantation of the acetabular component has been improved by the development of operation instruments, including the component holder and component pusher, which reduce the initial porosity of the bone cement. These lines of evidence also suggest that the mechanical properties of bone cement in vivo are strongly affected by the cementing techniques.

In conclusion, we found that the properties of bone cement were determined by the porosity of the cement but were not affected by the length of the period of implantation. The chemical structure of CMW1 cement was stable in vivo even after more than 20 years.
